# CTx001 for Geographic Atrophy: A Gene Therapy Expressing Soluble, Truncated Complement Receptor 1 (Mini-CR1)

**DOI:** 10.1016/j.xops.2025.100980

**Published:** 2025-10-21

**Authors:** Sonika Rathi, Athanasios Didangelos, Sofiya Pisarenka, Rachel Green, Stamatia Zafeiri, Peter Emery-Billcliff, Nakul Patel, Pamela Whalley, Parisa Zamiri, Viranga Tilakaratna, Ewa Szula, Rafiq Hasan, Mustafa M. Munye, Richard D. Unwin, Paul N. Bishop, Dennis Keefe, Simon J. Clark

**Affiliations:** 1 Institute for Ophthalmic Research, Eberhard Karls University of Tübingen, Tübingen, Germany; 2 Klinik und Poliklinik für Augenheilkunde, Universitätsmediz, Rostock, Germany; 3 Complement Therapeutics, London, UK; 4 Future Biomanufacturing Research Hub, Manchester Institute of Biotechnology, The University of Manchester, Manchester, UK; 5 Charles River Laboratories, Keele, Newcastle, UK; 6 Division of Cancer Sciences, School of Medical Sciences, Faculty of Biology, Medicine and Health, University of Manchester, UK; 7 Division of Evolution, Infection and Genomics, School of Biological Sciences, Faculty of Biology, Medicine and Health, University of Manchester, Manchester, UK; 8 University Eye Clinic, Eberhard Karls University of Tübingen, Tübingen, Germany; 9 Lydia Becker Institute of Immunology and Inflammation, Faculty of Biology, Medicine, and Health, University of Manchester, Manchester, UK

**Keywords:** AAV, Complement, Gene therapy, Macular degeneration, AMD

## Abstract

**Purpose:**

Preclinical evaluation of a novel gene therapy called CTx001 for treating geographic atrophy (GA). CTx001 encodes a protein called mini-CR1, which is a soluble fragment of complement receptor 1.

**Design:**

CTx001 was used in vitro and in vivo to analyze expression and complement-modulating activity. Mini-CR1 was used in vitro to analyze complement-modulating activity, and its ability to cross human Bruch’s membrane was evaluated ex vivo.

**Participants:**

CTx001, which is a self-complementary rAAV2 gene therapy vector expressing mini-CR1. Recombinant mini-CR1 protein, retinal pigment epithelium (RPE) cell lines, serum, human donor Bruch’s membrane, and a rat model.

**Methods:**

Recombinant mini-CR1 protein was produced in mammalian cells and purified. C3b and C4b breakdown assays were performed. Wieslab assays measured complement regulatory activity in serum. Mini-CR1 binding to C3b was measured using biolayer interferometry. The diffusion of mini-CR1 across human Bruch’s membrane was assessed using an Ussing chamber. Retinal pigment epithelium cell lines were transduced with CTx001 to assess expression, including directionality and complement modulatory activity. In vivo efficacy of CTx001 was tested using a rat laser–induced choroidal neovascularization (CNV) model.

**Main Outcome Measures:**

C3b/iC3b/C4b degradation, inhibition of membrane attack complex (MAC) formation in human serum, mini-CR1 binding to C3b, vector transduction efficiency, protein secretion and localization, and complement inhibition in vivo.

**Results:**

Mini-CR1 demonstrated potent cofactor activity for factor I–mediated cleavage of C3b, iC3b, and C4b; therefore, it inhibits both the alternative and classical complement pathways. It inhibited complement activation with an IC_50_ of 125 nM in human serum. Mini-CR1 demonstrated high-affinity binding to C3b. The mini-CR1 protein diffused across human Bruch’s membrane and retained activity postdiffusion. CTx001-transduced RPE cells secreted mini-CR1 apically and basolaterally, leading to reduced C3 activation and MAC deposition. In rats, subretinal administration of CTx001 resulted in a 75.4% reduction in MAC deposition in CNV lesions (*P* < 0.01).

**Conclusions:**

CTx001 is a potent inhibitor of complement. It efficiently transduces RPE cells, resulting in apical and basolateral secretion and crosses Bruch’s membrane, so it is expected to deliver mini-CR1 to the retina and choroid. These findings support its further development as a 1-time gene therapy for addressing complement overactivation in GA.

**Financial Disclosure(s):**

Proprietary or commercial disclosure may be found in the Footnotes and Disclosures at the end of this article.

Age-related macular degeneration (AMD) is a degenerative blinding disease that manifests itself through the progressive destruction of the macula, the central region of the retina responsible for high-resolution vision. The condition, which constitutes the leading cause of blindness in developed countries,^1^^,^^2^ has 2 late-stage forms: wet AMD, characterized by the formation of choroidal neovascularization (CNV) and geographic atrophy (GA; also called late dry AMD), in which patches of retinal cell death form. Since the introduction of anti-VEGF therapies as treatments for CNV, the wet form of AMD has been clinically well-managed.^3^ In 2023, 2 treatments for GA that target the complement system, called pegcetacoplan and avacincaptad pegol^4^ were approved by the US Food and Drug Administration. These therapies, which are delivered by regular intravitreal injection, inhibit complement and have been shown to slow the progression of GA lesions.

Evidence for the involvement of the complement system in AMD has emerged over nearly 2 decades of genetic, biochemical, and human tissue studies. The retina (neurosensory retina and retinal pigment epithelium [RPE]), along with the underlying Bruch’s membrane and choriocapillaris (the outer retinal blood supply), is where tissue damage occurs in AMD, and there is evidence for complement-mediated damage in all these layers (Fig 1A).^5^ Recent evidence has highlighted the role of complement overactivation in the choriocapillaris layer, particularly in the extracellular matrix (ECM) surrounding this layer of capillaries and its associated Bruch’s membrane.^6^, ^7^, ^8^, ^9^

**Figure 1:**
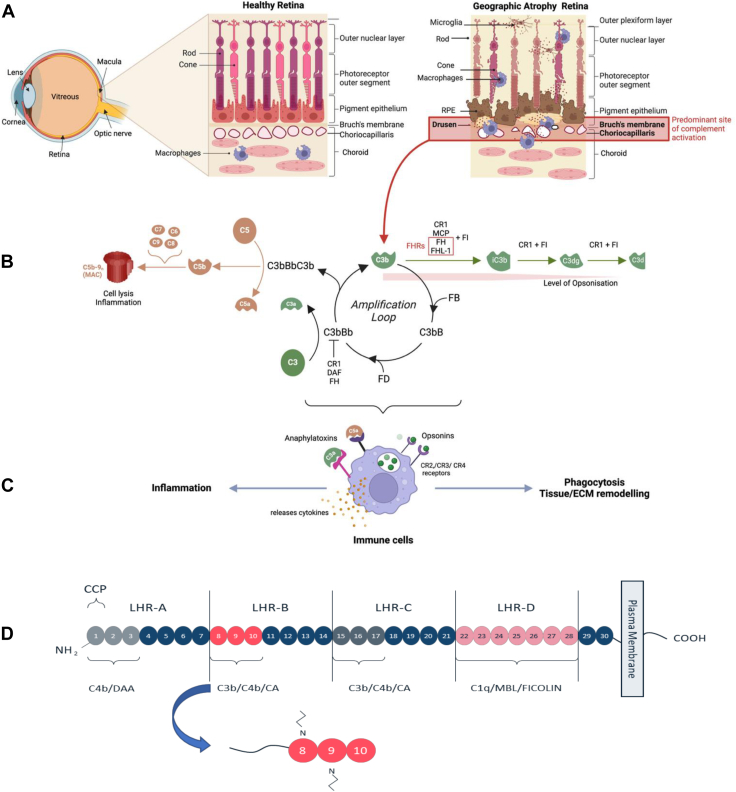
Schematic representation of the human eye, with a focus on retinal changes in geographic atrophy (GA) and complement activation. **A**, The human retina in the posterior part of the eye consists of the light-sensitive neurosensory retina and retinal pigment epithelium (RPE); underlying the RPE is Bruch’s membrane and the inner layer of the choroid, called the choriocapillaris. Geographic atrophy occurs in the central part of the retina called the macula. During the formation of GA lesions, which often form in areas with extracellular deposits in Bruch’s membrane called drusen, there is atrophy of the RPE, photoreceptors, and the choriocapillaris. This microenvironment is characterized by the presence of activated complement products in and around the ECM of Bruch’s membrane and the choriocapillaris that promotes both inflammation and the ingression of cells, including macrophages and microglia. **B**, Complement can be activated through 3 separate pathways,^5^ but the central protagonist remains the deposition of C3b onto cells and ECM. The binding of factor B (FB) and processing by factor D (FD) creates a C3 convertase (C3bBb), which enzymatically cleaves circulating C3 into C3b, feeding into the amplification loop. Subsequently, a C5 convertase is also formed (C3bBbC3b), which cleaves circulating C5 into C5b and triggers the formation of the membrane attack complex (MAC or C5b-9_n_) that causes cell lysis and contributes to a proinflammatory environment. Processing of C3 and C5 also produces C3a and C5a: 2 potent anaphylatoxins, which act as chemoattractants that recruit circulating immune cells to the site of complement activation. Complement activation is regulated by the protease factor I (FI), and this requires one of several cofactors, including factor H (FH), factor H-like protein 1 (FHL-1), membrane cofactor protein (MCP), and complement receptor 1 (CR1). Increasing concentrations of FHR proteins can interfere with the cofactor activity of FH and FHL-1 (red box). Together with a cofactor, FI cleaves C3b into inactive C3b (iC3b), which can no longer contribute to the amplification loop of complement activation. Both C3b and iC3b are potent opsonins: molecules that label a surface for phagocytosis by the immune cells recruited by the C3a/C5a anaphylatoxins. Opsonization is finally switched off through further degradation of iC3b by FI into C3dg and eventually C3d: CR1 is the only FI cofactor able to support this step. **C**, Engagement of the C3a or C5a receptors on recruited immune cells stimulates degranulation and release of proteases and proinflammatory cytokines. Opsonin engagement with specific receptors initiates phagocytosis and tissue/ECM remodeling. **D**, Native CR1 is a transmembrane glycoprotein comprised of 30 complement control protein (CCP) domains organized across 4 long homologous repeats (LHRs). Mini-CR1 comprises CCPs 8-10 (depicted in red), which contain the C3b/C4b binding domain, the preprotein form has an N-terminal signal peptide to enable secretion from cells. CA = cofactor activity; DAA = decay-accelerating activity; ECM = extracellular matrix; N = N-linked glycosylation sites.

The complement system consists of the classical, lectin, and alternative pathways that all feed into a central C3 amplification loop. The C3 amplification loop feeds into the C5 amplification loop, which in turn activates the terminal pathway and membrane attack complex (MAC) formation (Fig 1B). In the C3 amplification loop, C3 is broken down into C3a and C3b. C3b binds to factor B to form a convertase, which in turn stimulates more C3b production and downstream activation of the complement pathway via C5 cleavage, resulting in C5a production and MAC (C5b9n) formation. The anaphylotoxins C3a and C5a are potent mediators of inflammation and activators of immune cells (Fig 1C). Pegcetacoplan and avacincaptad pegol act by inhibiting the breakdown of C3 into C3a and C3b and C5 into C5a and C5b, respectively.

The C3 amplification loop is downregulated by the breakdown of C3b by the enzyme complement factor I (FI) into iC3b, a process that requires the presence of a FI cofactor. Cofactors include the soluble proteins factor H (FH) and its truncated variant factor H-like protein (FHL-1) and the cell membrane–bound complement receptor 1 (CR1). Factor H and FHL-1 allow the breakdown of C3b as far as iC3b, but this breakdown product remains a potent opsonin. In contrast, CR1 permits the further FI-mediated degradation of iC3b into C3c/C3dg toward lesser opsonin potency, a reduction in proinflammatory stimuli, and greater inactivation. Variations in the *CFH* gene that result in decreased function of the FH and FHL-1 proteins represent a major genetic risk factor for AMD. In addition, there are 5 FHR proteins (FHR1-5) that inhibit the actions of FH and FHL-1, thereby impeding C3b breakdown and driving complement activation; variations in and around genes encoding the FHR proteins, including *CFHR1*, *CFHR2*, *CFHR4,* and *CFHR5*, have been shown to be associated with, and indeed causal in, AMD.^10^, ^11^, ^12^, ^13^, ^14^

To develop a treatment addressing complement overactivation in GA, we elected to use the approach of enhancing cofactor activity, thereby generally suppressing complement activation and specifically restoring deficient cofactor activity in patients. We based our approach on CR1 because it has potent cofactor activity and is the only natural complement regulator capable of degrading iC3b. However, the native CR1 protein is a large (∼220 kDa) glycoprotein comprising 30 complement control protein domains (Fig 1D), whereas we aimed to use a small, secreted protein that can traverse Bruch's membrane and thereby treat complement overactivation on both the retinal and choriocapillaris sides of Bruch’s membrane. Therefore, we elected to use 1 of the 2 elements within CR1 that provide its cofactor activity, i.e., complement control proteins 8–10. Delivery of this protein using subretinal gene therapy has the advantage of being a potential one-off treatment rather than the frequent intravitreal injections required for current therapies. In addition, production and secretion of the protein from RPE cells could help avoid the blood–retina barrier and support effective distribution to both the retinal and choroidal compartments. To allow the protein to be secreted, the FH signal peptide was added, and the resultant therapeutic protein was called mini-CR1 (Fig 1D). An rAAV2 self-complementary vector was developed that encoded mini-CR1 (CTx001), and the biological activity and therapeutic potential of this vector and mini-CR1 are investigated as follows.

## Methods

### Ethics Statement

The requirement for informed consent and Institutional Review Board approval is waived for this study as no humans, nor human samples, were used. The anonymized human-derived iPSC-RPE cells were obtained commercially and are exempt from Institutional Review Board approval. All work described within this study is in adherence to the Declaration of Helsinki.

### Purified Recombinant Mini-CR1 Protein Production in HEK293T Cells

To produce purified, recombinant mini-CR1 protein, cDNA encoding complement control proteins 8-10 of CR1, which includes a TEV-cleavable N-terminal 6xHis tag, was incorporated into a pcDNA3.1 vector and transfected into HEK293T cells using previously described expression protocols.^15^ Further details on the expression and purification of recombinant mini-CR1 are provided in the Method S1 (available at www.ophthalmologyscience.org).

### C3b/C4b Degradation Assays

In vitro digestions of human C3b and C4b were conducted to assess the cofactor activity of mini-CR1 in the presence of human FI, and the results were analyzed by densitometry of chemiluminescence following Western blotting; see Method S1 (available at www.ophthalmologyscience.org) for further details.

### Alternative Complement Pathway Inhibition Assays

See Method S1 (available at www.ophthalmologyscience.org) for details.

### Binding Affinity Studies Using Biolayer Interferometry

Measurements of binding affinities for mini-CR1, FHL-1, and FH to C3b were performed using an Octet RH384 Biolayer Interferometry system (Sartorius) with biotinylated C3b immobilized onto streptavidin biosensors.^16^ For further details, see Method S1 (available at www.ophthalmologyscience.org).

### Ussing Chamber Diffusion

The diffusion properties of mini-CR1 across Bruch’s membrane were characterized ex vivo in Ussing chambers (Harvard Apparatus) with enriched human Bruch’s membrane as described previously.^17^ For further details, see Method S1 (available at www.ophthalmologyscience.org).

### rAAV Production and Transduction of RPE Cell Lines

rAAV constructs were prepared by SignaGen Laboratories using their proprietary rAAV Production Service. To assess the efficiency of rAAV constructs in expressing mini-CR1, transduction experiments were conducted in ARPE19, Htert-RPE1, and primary RPE cells. For further details of RPE cell transduction and analysis of expression, see Method S1 (available at www.ophthalmologyscience.org).

### Human iPSC-RPE Culture, Transduction, and Analysis of Directionality of Mini-CR1 Secretion

Human-induced pluripotent stem cell (iPSC)–derived RPE cells were cultured and then seeded onto Matrigel-coated transwell-12 inserts. They were allowed to form a monolayer, and tight junctions were assessed using occludin and ZO-1 immunostaining. Secretion of mini-CR1 into the apical and basolateral facing media was measured using a mesoscale electrochemiluminescence (Meso Scale Discovery [MSD] assay). See Method S1 (available at www.ophthalmologyscience.org) for further details.

### In Vivo Rat Studies

See Method S1 (available at www.ophthalmologyscience.org) for details.

## Results

### Mini-CR1 Drives Proteolysis of C3b, iC3b, and C4b by FI

Recombinant mini-CR1 is efficiently secreted by mammalian cells, and once purified, appears as 2 bands on reducing sodium dodecyl sulfate polyacrylamide gel electrophoresis gels (Fig S1, available at www.ophthalmologyscience.org). This dual band appearance is due to the presence of 2 N-glycosylation sites in mini-CR1, with the higher molecular weight band having both occupied, whereas the lower band only has 1 occupied: the 2 bands resolve to a single band after treatment with PNGase F to remove glycosylation (Fig S1).

**Figure 2:**
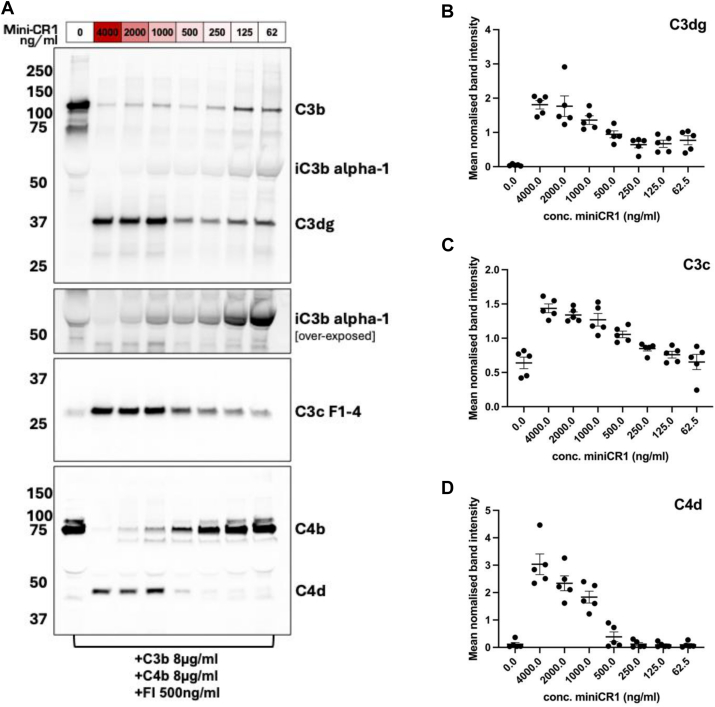
Recombinant mini-CR1 cleaves both C3b and C4b. **A**, Western blots assessing the bioactivity of mini-CR1 in vitro against high concentrations of human C3b 8 μg/mL and C4b 8 μg/mL, using a serial dilution of mini-CR1 ranging from 4000 to 62 ng/mL (133–2.1nM). A dose-dependent decrease in C3b, iC3b, and C4b, with a corresponding dose-dependent increase in their respective breakdown products, is shown: representative images of 5 separate experiments. Normalized densitometry values are plotted for the breakdown products C3dg (**B**), C3c (**C**), and C4d (**D**), showing the cumulative data from all 5 experiments (n = 5) ± SD. CR1 = complement receptor 1; SD = standard deviation.

C3b breakdown assays demonstrated that mini-CR1 at concentrations as low as 62 ng/mL (2.1 nM) caused substantial consumption of C3b with the production of iC3b, C3dg, and C3c (Fig 2A–C). Because the concentration of mini-CR1 increased, the consumption of C3b became more complete, as did the breakdown of iC3b to C3dg and C3c. C4b degradation also occurred in a dose-dependent pattern with increasing doses of mini-CR1, resulting in decreased C4b and an increase in C4d fragments, although the digestion efficiency was lower than for C3b (Fig 2A, D). In contrast to mini-CR1 and CR1, other FI cofactors only facilitate the cleavage of C3b into iC3b. This is demonstrated for FHL-1 (Fig S2, available at www.ophthalmologyscience.org) and known to be the case with FH and CD46/MCP.^18^ Mini-CR1 shares the same capacity as soluble CR1 (a shortened form of CR1 that lacks a cell membrane binding domain found in very low concentration in blood) to drive the breakdown of C3b into C3dg (Fig S2).

**Figure 3:**
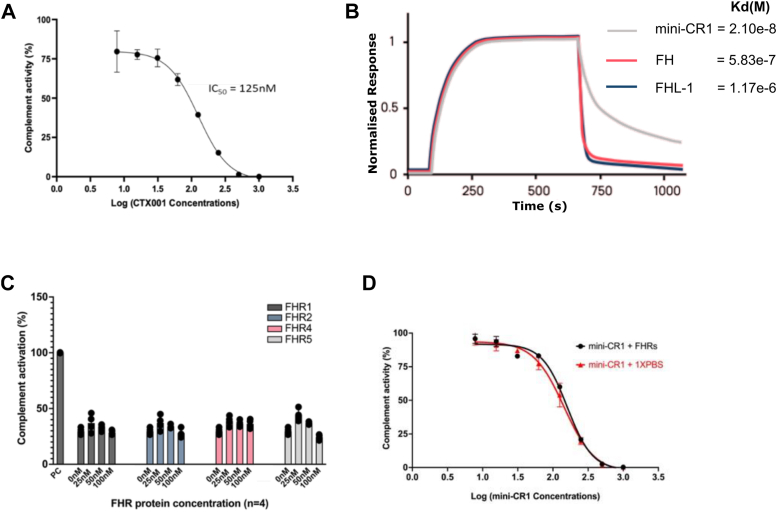
Further characterization of mini-CR1 activity. **A**, Wieslab assay performed on zymosan-activated human serum in the presence of increasing concentrations of mini-CR1. Mean values (SD) are presented for 3 independent experiments. **B**, Biolayer interferometry analysis of binding of fluid-phase mini-CR1, FH, and FHL-1 to immobilized C3b, demonstrating that mini-CR1 binds with the highest affinity. **C**, Complement activation levels in activated human serum (Wieslab assay) at fixed mini-CR1 concentration (161.7 nM) and increasing levels of supplemented FHR proteins. Mini-CR1 cofactor activity is not inhibited by any singular FHR protein tested, even at supraphysiological concentrations. Mean values (SD) are presented, where PC = positive control (human serum without CTx001 and without addition of FHRs). **D**, Wieslab assay of complement alternative pathway activation in the presence of increasing levels of mini-CR1 and the presence (red) or absence (black) of physiological levels of FHR proteins: FHR1, 38 nM; FHR2, 55 nM; FHR4, 54 nM; and FHR5, 28 nM (29). IC_50_ in the absence of FHR = 143.9 nM; IC_50_ in the presence of FHR = 159.8nM, where data is shown as mean (SD). CR1= complement receptor 1; FH = factor H; FHL-1 = factor H-like protein; SD = standard deviation.

Cleavage of C3b into iC3b and beyond by mini-CR1 would effectively inhibit the continuation of the amplification loop of the complement cascade. To confirm this, we used the alternative pathway Wieslab assay to assess the inhibitory effects on complement turnover in human serum; this was demonstrated with an IC_50_ of 125 nM with mini-CR1 (Fig 3A). Similarly, we tested serum from other species and saw for cynomolgus monkey serum an IC_50_ of 26 nM and rat serum 165 nM (Fig S3, available at www.ophthalmologyscience.org). The binding affinity of mini-CR1 to immobilized C3b investigated by BLI was *Kd* = 2.1 × 10^–8^ M, stronger than for both FH (*Kd* 5.83 × 10^–7^ M) and FHL-1 (*Kd* 1.17 × 10^–6^ M); therefore, mini-CR1 has a higher affinity for the C3b protein than other cofactors (Fig 3B). This replicates other studies that have found the C3b-binding regions of CR1 to have high C3b-binding affinities,^19^^,^^20^ and it is believed that this is the mechanism by which CR1 can drive the degradation of C3b by FI beyond iC3b.^21^

### Mini-CR1 Is Unaffected by Increased Levels of FHR Proteins

FHR proteins have been demonstrated to be elevated in the circulation and retinal tissue in AMD, and so provide a unique environment and an additional challenge for driving C3b breakdown by modifying cofactor activity. Supplementation of individual purified recombinant FHR proteins 1, 2, 4, and 5 (expressed and purified as described previously^11^) into human serum to supraphysiological levels failed to interfere with the inhibitory effects of mini-CR1 on complement activation (Fig 3C). Similarly, when these FHR proteins were pooled and supplemented into human sera at levels matching the elevated concentrations found in patients with AMD from previous studies,^10^^,^^12^ there was no effect on the IC_50_ value of mini-CR1, as shown in Figure 3D.

### Mini-CR1 can Diffuse Through Human Bruch’s Membrane

**Figure 4:**
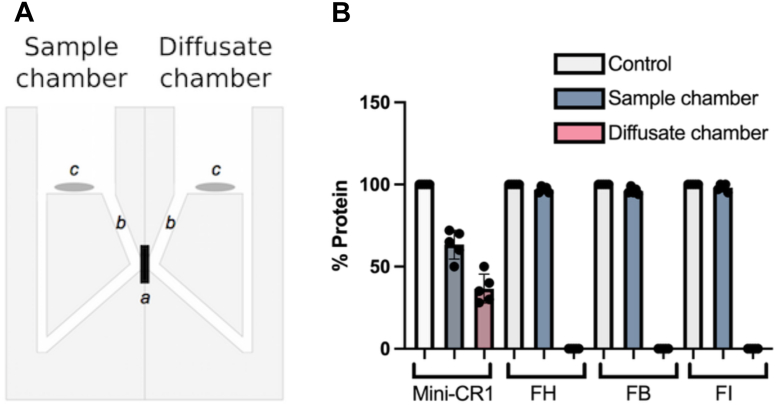
Bruch’s membrane diffusion properties of mini-CR1. **A**, Schematic representation of an Ussing chamber used in diffusion experiments, where a: enriched macula Bruch’s membrane; b: sampling access points; and c: magnetic stirrers. Recombinant mini-CR1, FH, FB, or FI were placed in the sample chamber, and after 24 hours, the presence of protein in both chambers was assessed by SDS-PAGE. **B**, Summary analysis of diffusion experiments (Bruch’s membrane from n = 5 AMD donors) analyzed by SDS-PAGE showing that mini-CR1 is the only protein tested able to passively diffuse across Bruch’s membrane: mean (SD) depicted in the bar chart with individual data points shown. AMD = age-related macular degeneration; CR1= complement receptor 1; FB = factor B; FH = factor H; FI = factor I; SD = standard deviation; SDS-PAGE = sodium dodecyl sulfate polyacrylamide gel electrophoresis.

Complement turnover and MAC deposition associated with AMD occur particularly on and around the ECM of the choriocapillaris layer underlying the retina.^6^, ^7^, ^8^, ^9^ Bruch’s membrane alone, even without the retina, can present a barrier to therapeutics delivered to the retina (or vitreous humor) reaching the choriocapillaris. Therefore, we tested whether mini-CR1 can traverse Bruch’s membrane. Bruch’s membrane was enriched from 5 postmortem human donor eyes over the age of 65 years using previously described protocols^22^ and set within an Ussing chamber,^17^ which allows the passive diffusion of proteins from 1 chamber (sample chamber) into the other chamber (diffusate chamber) through the membrane being tested (Fig 4A). After 24 hours, for all 5 Bruch’s membrane samples, mini-CR1 is found in both chambers after passive diffusion through Bruch’s membrane, whereas other proteins tested, including FH, FI, and factor B, were not detected in the diffusate chamber after 24 hours (Fig 4B), as has previously been observed.^17^ C3b breakdown assays confirmed that mini-CR1 maintains its cofactor activity after crossing Bruch’s membrane, whereas pegcetacoplan (APL-2) struggles to diffuse through this tightly packed membrane.^23^

### Vector Design for rAAV Delivery of Mini-CR1 to RPE Cells

**Figure 5:**
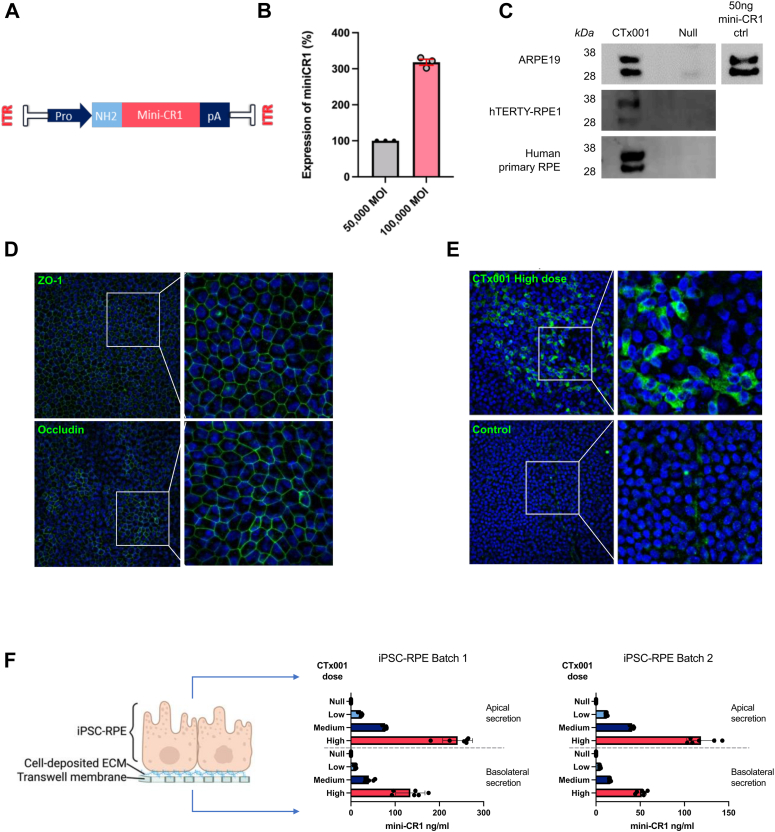
Transduction efficiency of CTx001 in RPE cell lines. **A**, Schematic of CTx001, a self-complementary rAAV2 expressing the mini-CR1 transgene with an N-terminal secretory leader sequence. ITR = Inverted terminal repeat; NH2 = Leader sequence; pA = Poly A sequence; Pro = Promoter. **B**, Comparative analysis of transduction efficiency of the rAAV2 delivered CTx001 in driving mini-CR1 expression at 50 000 and 100 000 MOI in hTERT-RPE1 cells evaluated by quantitative reverse transcription polymerase chain reaction, 72 hours posttransduction. **C**, mini-CR1 expression and secretion from transduced human ARPE19 cells, hTERT-RPE1 cells, and primary human RPE cells as assessed by Western blotting of culture supernatants. **D**, Quality control staining for iPSC-RPE tight-junction formation showing both ZO-1 and occludin. **E**, Staining iPSC-RPE cells for mini-CR1 (green) after transduction with a high dose of CTx001 (i.e., 2.2 × 10^10^ vg/transwell). **F**, Bidirectional secretion of mini-CR1 from iPSC-RPE cells 7 days posttransduction with CTx001 at one of 3 different doses: low, 9 × 10^8^ vg/transwell (∼8000 MOI); medium, 4.5 × 10^9^ vg/transwell (∼40 000 MOI); or high, 2.2 × 10^10^ vg/transwell (∼200 000 MOI); compared with transduction with empty capsid (null) at 3.4 × 10^10^ vg/transwell as a control; mini-CR1 levels were measured using an MSD assay. The results from 2 separate batches of iPSC-RPE are shown. CR1= complement receptor 1; iPSC = induced pluripotent stem cell; MOI = multiplicity of infection; MSD = Meso Scale Discovery; RPE = retinal pigment epithelium.

Given the challenges associated with frequent intravitreal injections for patients with GA, we explored a gene therapy approach to give sustained delivery of mini-CR1. To achieve this, we designed and tested a series of different constructs for their ability to transduce the RPE and maximize mini-CR1 expression. We used in vitro and in vivo approaches (human RPE cell lines and naïve C57BL/6JRj mice undergoing subretinal injection, data not shown) and on this basis took forward for further study a self-complementary design with rAAV2 serotype, already known to effectively target RPE cells,^24^ using a ubiquitous promoter and the secretory leader sequence from FH (Fig 5A). CTx001, the self-complementary rAAV2-vectorized mini-CR1, showed a dose-dependent increase in mini-CR1 transcription in RPE cells by quantitative reverse transcription polymerase chain reaction (Fig 5B). Subsequent transduction analysis using CTx001 at 100 000 multiplicity of infection on both RPE cell lines (hTERT-RPE1 and ARPE19) and human primary RPE showed mini-CR1 gene transcription by quantitative reverse transcription polymerase chain reaction (data not shown), and secretion of the mini-CR1 protein into the culture supernatant by Western blot analysis using a rabbit polyclonal antibody against mini-CR1 (Fig 5C).

To investigate further the expression and secretion of mini-CR1 by RPE cells, we employed iPSC-RPE that were fully differentiated in a transwell setting; these formed a confluent monolayer and expressed a series of RPE cells’ tight-junction markers, including occludin and ZO-1 (Fig 5D). These iPSC-RPE cells were transduced with 3 separate doses of CTx001 (low, 9 × 10^8^ vg/transwell; medium, 4.5 × 10^9^ vg/transwell; and high, 2.2 × 10^10^ vg/transwell); after 7 days, mini-CR1 staining was performed (Fig 5E), and samples were taken from both the apical and basolateral chambers of the transwell system in which the cells were grown. Meso Scale Discovery assay showed secretion of mini-CR1 from transduced iPSC-RPE cells both apically and basolaterally (60% and 40% total secreted protein, respectively), through the cell-deposited ECM and the transwell membrane itself (Fig 5F), thereby predicting effective delivery of mini-CR1 to both the neurosensory retina and through Bruch’s membrane to the choriocapillaris.

### CTx001 Delivered Mini-CR1 Shows Activity in RPE Cell Line

ARPE19 cells were transduced with CTx001 (1.78 vg/cell) and maintained for 7 days to allow stable expression, secretion, and accumulation of mini-CR1 transgene protein. Cells were then incubated with 10% normal human serum to provide all necessary complement components for activation. Complement activation was triggered by the addition of 100 μg/mL zymosan and 10 μg/mL lipopolysaccharide (LPS) for 16 hours (to represent the long-term delivery of mini-CR1). Lipopolysaccharide and zymosan promote complement activation by providing surfaces that facilitate C3b deposition and subsequent C5b9 formation: zymosan activates the alternative pathway by serving as a binding surface for C3b, leading to amplification of the cascade,^25^ and LPS engages both the classical and alternative pathways, further enhancing complement activation.^26^ In both pathways, C3b and C4b contribute to the formation of C5 convertases (C3bBbC3b and C4b2aC3b), which cleave C5 into C5a and C5b. C5b then sequentially binds C6, C7, C8, and multiple C9 molecules, forming MAC, which mediates cell lysis. Mini-CR1, as a cofactor for FI, should augment C3b and C4b proteolysis, reducing C5 convertase formation and thereby limiting C5 cleavage and subsequent MAC assembly.

**Figure 6:**
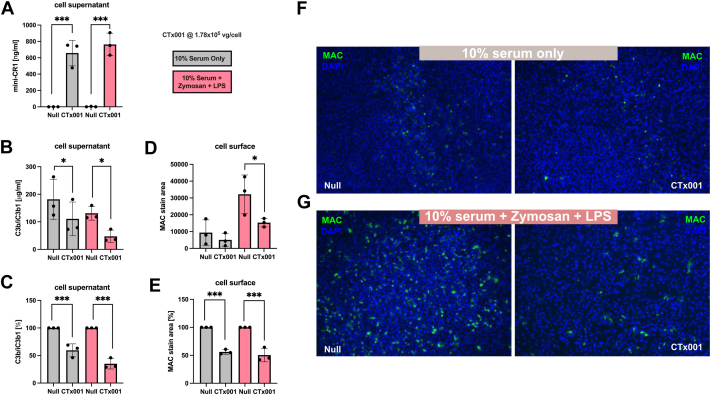
In vitro evaluation of mini-CR1 in RPE cell line transduced with CTx001. ARPE19 cells were transduced with CTx001 AAV2 at 1.78E5vg/cell and maintained for 7 days in culture to allow for mini-CR1 transgene expression and protein accumulation in the culture medium. Control ARPE19 cells were incubated for 7 days with Null AAV2 (empty capsids) at 1.75E5vp/cell. **A**, Soluble mini-CR1 was detected in the cell supernatants from ARPE19 cells incubated in either 10% normal human serum, or 10% normal human serum where complement had been activated by the presence of zymosan (100 μg/m) and LPS (10 μg/mL). **B, C**, The increase in mini-CR1 expression was accompanied by a significant decrease in C3b/iC3b levels in both normal and complement-activated serum. **D, E**, Membrane attack complex deposition on the surface of the ARPE19 cells was detected using a neoepitope-specific anti-C9 antibody (clone aE11). A significant decrease in MAC deposition on the cell surface is observed in the cells cultured with complement-activated serum with cells transduced with CTx001. **F, G**, representative images of the MAC staining on cells cultured in either normal human serum (**F**) or complement-activated serum (**G**). For each data set, n = 3, and bar graphs are plotted mean ± SD. Statistical comparisons were performed using ANOVA followed by Bonferroni post hoc test to detect differences between control and CTx001-treated cells. ∗ *P < 0.*05 represents and ∗∗∗ *P < 0.*005. ANOVA = analysis of variance; CR1= complement receptor 1; DAPI = 4′,6-diamidino-2-phenylindole; LPS = lipopolysaccharide; MAC = membrane attack complex; RPE = retinal pigment epithelium; SD = standard deviation.

CTx001-AAV2–induced mini-CR1 expression in ARPE19 cells resulted in ∼700 ng/mL (∼24 nM) mini-CR1 in the supernatant as quantified by MSD assay (Fig 6A). Mini-CR1 secretion remained consistent regardless of the presence of human serum (gray bars) or serum supplemented with zymosan/LPS (red bars). The increase in mini-CR1 was accompanied by a significant reduction in C3b-iC3b1 levels in the supernatant (Fig 6B, C), measured using an MSD assay that first captures total C3b and then detects the 1H8 epitope, specific for C3b converted to iC3b.^27^ These findings indicate that mini-CR1 in the ARPE19 supernatant actively facilitates the consumption of C3b and iC3b1 derived from human serum.

Mini-CR1 bioactivity was further evaluated by staining for MAC on the surface of these serum- and zymosan-activated ARPE19 cells (Fig 6F, G). Membrane attack complex deposition was detected using an aE11 neoepitope-specific antibody, which recognizes C9 incorporated into MAC complexes.^28^ Incubation of ARPE19 cells with fresh human serum led to spontaneous accumulation of C9 neoepitope staining, reflecting MAC deposition on the cellular surface (Fig 6D, F). The addition of zymosan/LPS further increased C9 neoepitope staining, indicating enhanced MAC formation (Fig 6E, G). In CTx001-infected ARPE19 supernatants, where mini-CR1 reached ∼700 ng/mL, a significant reduction was observed in C9 neoepitope staining, measured as cell surface deposits. Because C3b and all necessary complement components for MAC formation were provided by the addition of human serum, these results indicate that mini-CR1 not only reduces C3b/iC3b1 levels but also limits MAC deposition on the cell surface.

### Subretinal Delivery of CTx001 in a Rat Laser–Induced CNV Model

Having demonstrated that mini-CR1 inhibits complement by driving the complete degradation of C3b and the degradation of C4b, and that it can be delivered successfully to RPE cells with gene therapy, we aimed to test its ability to suppress complement in vivo. Rodent laser–induced CNV models were originally optimized for the study of ocular vascular diseases^29^ and played a major role in the identification of VEGF as a target for ocular neovascularization.^30^ Although not a model for GA in AMD (of which none exist), the laser CNV model results in increased complement turnover and the deposition of MAC within the retina.^31^^,^^32^ As such, complement inhibitors can be tested in this model,^33^, ^34^, ^35^ using MAC deposition as a biomarker of complement turnover in the posterior part of the eye.

**Figure 7:**
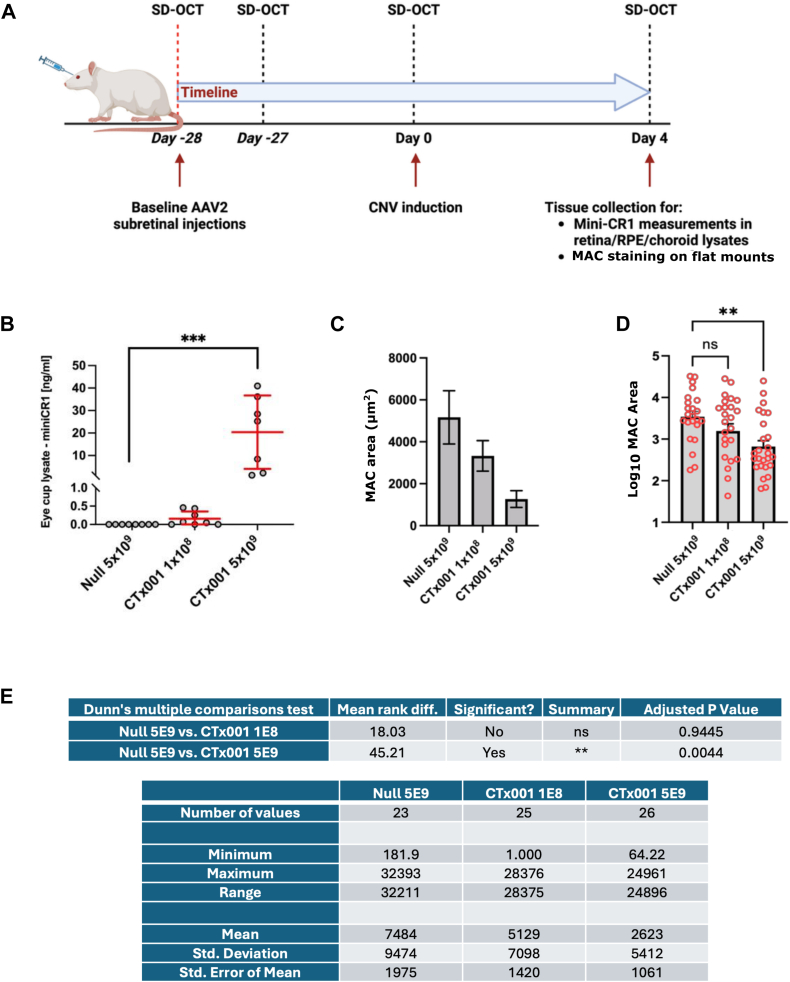
Verification of in vivo target engagement by CTx001 in the rat laser CNV model. **A**, Timeline schematic of the in vivo rat laser–induced CNV model used in this study. The number of rats and details of each group are listed in Supplementary Table 1 (available at www.ophthalmologyscience.org). Two different rAAV2 doses were injected as follows: low, 1 × 10^8^ vg/eye; and high, 5 × 10^9^ vg/eye, n = 9 rats per group. Control animals (n = 9) received null rAAV2 subretinal injections (at 5 × 10^9^ vg/eye). **B**, mini-CR1 protein expression was detected and quantified with a Meso Scale Discovery (MSD) immunoassay using eye-cup lysates. One-way ANOVA was performed (n = 8) ∗∗∗ *P* < 0.005. **C**, Membrane attack complex deposition was measured by immunofluorescence in flat-mounted eyecups treated with null or CTx001, where mean values ± SD are shown. Representative images depicting laser lesion as defined by phalloidin staining and MAC staining within the lesion region of interest after background threshold has been applied are shown in Figure S4 (available at www.ophthalmologyscience.org). The data set failed the Kolmogorov–Smirnov test for normality and was therefore transformed using log10(X). **D**, Log10 transformed data of the MAC staining showing mean ± SD values; ANOVA followed by Kruskal–Wallis post hoc test was performed on log10 transformed data and demonstrated that the high-dose mini-CR1 group had a significant reduction in MAC immunostaining. ∗∗ *P* < 0.01. **E**, Summary of statistical analysis and descriptive statistics used in (**D**). ANOVA = analysis of variance; CNV = choroidal neovascularization; CR1= complement receptor 1; MAC = membrane attack complex; RPE = retinal pigment epithelium; SD = standard deviation; SD-OCT = spectral-domain OCT.

It has been suggested that the mouse complement system does not behave in the same manner as that of human complement,^36^^,^^37^ and that, in fact, rat complement makes a far better model of the human system. Therefore, we performed the experiments using a rat laser–induced CNV model. Subretinally delivered CTx001 at 2 different doses, low (1 × 10^8^ vg/eye) and high (5 × 10^9^ vg/eye), were compared with null vector (5 × 10^9^ vg/eye), with 9 rats in each group (Fig 7A and Table S1, available at www.ophthalmologyscience.org). Eye-cup lysates were collected 28 days after subretinal injections, and using the MSD assay, a dose-dependent secretion of the mini-CR1 protein was observed (Fig 7B). A reduction of 75.4% in the level of MAC staining was observed in rat eyes treated with high-dose CTx001 compared with null vector alone (Fig 7C). Given that the raw data did not follow a normal distribution, direct analysis of variance analysis cannot be applied. However, log_10_ transformed data fulfill all of the tests for normality (Shapiro–Wilk, D'Agostino–Pearson, and Kolmogorov–Smirnoff), and thus analysis of variance analysis was applied and showed the 75.4% reduction to be statistically significant (*P* < 0.01; Fig 7D).

## Discussion

The first treatments approved for GA, pegcetacoplan and avacincaptad pegol, have validated the complement system as a therapeutic target. However, these require monthly or bimonthly intravitreal injections to maintain efficacy. This places a considerable burden on both patients and health care providers, and although they slow GA lesion growth, they have not yet been shown to improve functional outcomes in visual acuity. Therefore, we elected to develop a complement modifier for treating AMD, CTx001, that is delivered as a single surgical treatment and produces a sustained effect, and which targets multiple pathways involved in complement activation and turnover. CTx001 is a gene therapy designed to be delivered by subretinal injection (i.e., creation of a transient bleb between the neurosensory retina and RPE). As it uses an rAAV2 vector, it is expected to predominantly target the RPE,^38^ which then secretes the small, soluble complement inhibitor protein, mini-CR1, in a so-called biofactory approach.

We demonstrated that RPE cells transduced with CTx001 secrete mini-CR1 apically and basolaterally (Fig 5F). Furthermore, we demonstrate that it inhibits MAC formation on the surface of CTx001 transduced ARPE19 cells and promotes C3b consumption in the cell culture media (Fig 6). Using healthy human Bruch’s membrane ex vivo, we demonstrated that mini-CR1 crosses Bruch’s membrane (Fig 4); it is important to test this using human tissue because there is wide variation in Bruch’s membrane thickness and permeability between species.^39^ The ability of an AMD therapeutic to cross Bruch’s membrane is now recognized as an important consideration for therapeutic design and delivery modality selection.^40^^,^^41^ Bruch’s membrane acts as a selective barrier to many proteins, including complement proteins, based on size, hydrodynamic radius, and charge^17^^,^^42^; if complement therapies are delivered to the vitreous humor or retina, failure to cross Bruch’s membrane may pose a significant barrier to realizing their full efficacy potential, because their function will be limited to retinal tissues and they will not treat complement overactivation in the choriocapillaris layer. Therefore, we predict that the transduced RPE cells will continually secrete mini-CR1 that will reach both the retina and choroid, thereby inhibiting complement overactivation in all anatomic compartments where complement overactivation has been demonstrated in AMD. However, a potential limitation of this study is that we did not use Bruch’s membrane from donors with AMD, which may have different diffusion properties, especially at the macula, compared with the tissue we used from donors aged >65 years without AMD.

We have shown that mini-CR1 is a potent cofactor for the FI-mediated degradation of C3b, iC3b, and C4b, so it targets the alternative and classical complement pathways. The breakdown of C3b to iC3b removes it from the complement C3 amplification loop, thereby decreasing anaphylatoxin production and downstream MAC deposition. Mini-CR1 is an effective cofactor for FI and is more potent than other cofactors, including FH and FHL-1, so it can break down C3b beyond iC3b to C3dg. This can be explained by its higher affinity to C3b compared with FH and FHL-1 (Fig 3B). Although iC3b cannot contribute to the C3 amplification loop, it acts as a strong ligand for CR2, CR3, and CR4 receptors on immune cells, and the resultant interaction with immune cells results in continued opsonization and remains proinflammatory. The further degradation to C3dg by mini-CR1 removes, or greatly reduces, interactions with immune cells, thereby further reducing their potency and the proinflammatory microenvironment.

Recently, genetically driven elevated levels of circulating FHR proteins have been associated with AMD pathogenesis.^10^, ^11^, ^12^ Indeed, there is evidence from Mendelian randomization studies that high FHR protein levels cause AMD. These highly homologous proteins are closely related to the FH protein and share some of its binding partners.^43^ FHR proteins bind to C3b but cannot bind to FI, so they are not cofactors for FI-mediated C3b breakdown; instead, they can act as complement activators by outcompeting FH and FHL-1 binding to C3b, thereby decreasing C3b breakdown and causing excessive complement activation.^11^^,^^44^ Given that these FHR proteins accumulate in the ECM surrounding the choriocapillaris in human eyes, as well as in drusen within Bruch’s membrane,^11^^,^^12^^,^^45^ we assessed whether FHR proteins hinder the cofactor activity of mini-CR1. We demonstrated that mini-CR1 is not displaced by supraphysiological levels of FHR proteins in human serum, so mini-CR1 maintains cofactor activity for FI in the presence of FHR proteins (Fig 3D, E).

There are no satisfactory in vivo models of GA. However, in rodent laser CNV models, complement overactivation is observed. This occurs in the region of the retina and choroid, so it is in an anatomically relevant location. We used the rat laser CNV model, and the readout used for the effect of CTx001 was deposition of MAC. We demonstrated significantly reduced levels of MAC deposition after laser CNV induction (75% compared with null vector controls; Fig 7). Membrane attack complex deposition has also been used to evaluate soluble CD59, the native complement regulator that naturally targets MAC cellular deposition directly,^46^ as a potentially therapeutic complement modifier in the eye. In this case, MAC deposition in the laser-induced CNV mouse model is reduced by ∼50%.^33^ Other groups have used CNV lesion size and leakage as a readout, but we believe MAC is a more direct and specific readout of complement overactivation.

In summary, we present here a first-generation, self-complementary rAAV2-mediated gene therapy (CTx001) that successfully transduces RPE cells, resulting in secretion of the soluble complement modifier mini-CR1. This modifier can likely address both complement-meditated amplification and opsonization, in retinal tissues, Bruch’s membrane, and the choriocapillaris. This ‟one-shot and done” delivery approach was shown to be very efficacious in reducing complement turnover and can target the complement cascade at the levels of both C3b, iC3b, and C4b. CTx001 is expected to address the continued unmet need in the therapeutic landscape for the intervention of complement overactivation associated with GA.
