# Supplementary material for: CTx001 for Geographic Atrophy: A Gene Therapy Expressing Soluble, Truncated Complement Receptor 1 (Mini-CR1)
Source: Ophthalmol Sci. 2025 Oct 21;6(1):100980. doi: 10.1016/j.xops.2025.100980 (PMC12689202; doi:10.1016/j.xops.2025.100980)
Supplement: Supplemental material [file mmc1.pdf]

CTx001 for geographic atrophy: a gene therapy expressing soluble, truncated complement receptor 1 (mini-CR1)

Sonika Rathi[1], Athanasios Didangelos[2], Sofiya Pisarenka[2], Rachel Green[2], Peter Emery-Billcliff[2], Nakul Patel[2], Pamela Whalley[2], Parisa Zamira[2], Viranga Tilakaratna[3,†], Ewa Szula[4,†], Rafiq Hasan[2], Mustafa Munye[2], Richard D. Unwin[5], Paul N Bishop[6], Dennis Keefe[2,*], Simon J Clark[1,7,8,*]

[1]Institute for Ophthalmic Research, Eberhard Karls University of Tübingen, Tübingen, Germany; [2]Complement Therapeutics, London, UK; [3]Future Biomanufacturing Research Hub, Manchester Institute of Biotechnology, The University of Manchester, 131 Princess Street, Manchester, M1 7DN, UK; [4]Charles River Laboratories, Keele, Newcastle, UK; [5]Division of Cancer Sciences, School of Medical Sciences, Faculty of Biology, Medicine & Health, University of Manchester, UK; [6]Division of Evolution, Infection & Genomics, School of Biological Sciences, Faculty of Biology, Medicine & Health, University of Manchester, UK; [7]University Eye Clinic, Eberhard Karls University of Tübingen, Tübingen, Germany; [8]Lydia Becker Institute of Immunology and Inflammation, Faculty of Biology, Medicine, and Health, University of Manchester, Manchester, UK.

[†]Current contact address.

Supplementary Information

Supplementary Methods

Purified recombinant mini-CR1 protein production in HEK293T cells

1 mg/ml plasmid DNA and 7.5 mM PEI max transfection reagent (Polysciences, Germany) were added to separate aliquots of 150 mM NaCl and each incubated for 10 min at room temperature. The DNA/PEI mixture was added to a 15 cm diameter dishes containing 7×10^6 HEK293T cells/per dish in a dropwise manner and incubated at 37°C. After 5–6 hours, the media was replaced with 2% (v/v) FCS containing DMEM (no antibiotic) and incubated at 37°C for overnight. Conditioned media was collected daily and replaced with fresh media. Conditioned media from each of 20 dishes (17.5 ml/dish) were collected and pooled after 24, 48, 72, and 144 hours. The pooled conditioned media after 144 hours (1,400 ml total) was diluted with the addition of 600 ml 50 mM HEPES, 500 mM NaCl, 20 mM Imidazole, pH 7.5. To this, 12 ml of NiNTA resin (Expedeon) was added and incubated overnight with rotation at 4°C. The NiNTA beads were collected by passing the media through empty PD10 columns with a filter (GE Healthcare) by gravity flow (500 ml media per PD10 column, i.e. four PD10 columns in total). The beads were then washed with 10 ml of wash buffer (50 mM HEPES, 500 mM

NaCl, 20 mM imidazole, pH 7.5). Finally, the His-tagged mini-CR1 protein was eluted using 16 ml of elution buffer (50 mM HEPES, 500 mM NaCl, 500 mM imidazole, pH 7.5). Eluted protein was dialysed back into wash buffer overnight at 4°C before being concentrated further by the addition of 1.5 ml NiNTA beads and then eluted into 6 × 1 ml aliquots. Purified protein aliquots were dialysed into 20 mM glycine, 125 mM NaCl, pH 9.0 using a Slide-A-Lyzer dialysis cassette (Thermo Fisher Scientific, USA) with a 10 kDa cut off. The purified recombinant protein was assessed for purity by SDS-PAGE and visualised by staining the gels for 60 min at room temperature with Instant Blue Coomassie stain (Expedeon, UK).

C3b/C4b degradation assays

The assay mixture contained C3b (8 µg/mL), C4b (8 µg/mL), and FI (0.5 µg/mL), all from CompTech, USA, diluted in 1× PBS with 500 µg/mL BSA. Mini-CR1 was serially diluted from 4000 ng/mL (133 nM) to 62 ng/mL (2.1 nM). Reactions were incubated at 37 °C with shaking (800 rpm, Thermomix) for 16 hours (x2 predicted half-life of mini-CR1) to model the sustained exposure expected with *in vivo* gene therapy approach. Following incubation, samples were prepared for immunoblotting. A total of 120 µL from each reaction was mixed with 40 µL of 4× Laemmli SDS sample buffer, boiled at 95 °C for 10 minutes, and 25 µL was loaded per lane. Proteins were separated on Bolt™ Bis-Tris 4–12% protein gels and transferred nitrocellulose membranes. Membranes were blocked in 10% fat-free milk powder dissolved in 1× PBS with 0.05% Tween-20 (PBS-T) for at least 60 minutes, then incubated overnight at 4 °C with primary antibodies. The following primary antibodies were used: rabbit anti-C3d (Dako), rabbit anti-C4d (Abcam), and mouse anti-C3c clone F1-4 (Hycult), each diluted 1:500 in 5% BSA in 1× PBS. After primary incubation, membranes were washed four times for 10 minutes each with PBS-T. HRP-conjugated swine anti-rabbit IgG (1:2000 in 5% BSA) was applied for 65 minutes, followed by four additional washes. HRP signals were detected using ECL-Plus reagents (2-minute incubation) and visualized on the iBright 1500 imaging system. Immunoblots were quantified by densitometry of chemiluminescence signals using ImageJ for C3b, C3dg, C3c, C4b and C4d. For each of the five independent C3b/C4b digestions and corresponding immunoblots, raw density values were scaled by mean normalization. Specifically, each lane's value was normalized to the average densitometry value of all lanes within the same blot.

Alternative complement pathway inhibition assays

The Wieslab Alternative Complement Pathway Assay (SVAR COMPLAP330, Sweden) was employed to assess the mini-CR1 complement inhibitory activity in human and Cynomolgus Monkey serum and the Hycult Alternative Complement Pathway Assay (Hycult Biotech, The Netherlands) was employed for murine serum analysis. Mini-CR1 protein at concentrations ranging from 1000 nM to 7.8135 nM was

prepared via 8-fold serial dilution in 200 µL of the assay buffer. For the positive control (PC) Zymosan activated human serum and cynomolgus monkey serum (diluted 1:18) and rat serum (diluted 1:5) without the addition of mini-CR1 were used, while dilution buffer alone or serum without complement activity were used as the negative control (NC). Subsequently, 100 µl of each (1000 nM to 7.8135 nM) mini-CR1 protein was added to 100 µl of 1:18 pre-diluted activated human serum and cynomolgus monkey serum and 1:5 pre-diluted rat serum in the assay diluent and allowed to incubate for 30 minutes at RT. After incubation samples, PC, NC, and diluent alone (Blank) were transferred to the assay plate in duplicate and further incubated for 1 hour at 37°C followed by washing three times with the 1x wash buffer. Next, 100 µL of the conjugate/tracer, was added to each well and incubated for 30 min at RT. The conjugate in the Wieslab assay consists of an alkaline phosphatase-conjugated antibody targeting the primate C5b-9 neoantigen, while the Hycult assay uses a biotinylated antibody specific to rat C5b-9. Therefore, for detection of the bound antibodies in the Weislab assay, an alkaline phosphatase substrate solution (PnPP) was used, 100 µL in each well, and incubated for 30 min at RT. While in the Hycult assay, 100 µL of streptavidin peroxidase solution was added, incubated for 30 min at RT followed by washing and 100 µL of TMB in each well. After 30 mins incubation in the dark, the reaction was stopped by the addition of oxalic acid solution. The amount of complement activation was measured by the absorbance at 405 nm (Weislab assay) or 450 nm (Hycult assay) using a Molecular Devices VersaMax Microplate Reader (Marshal Scientific, USA). The results were expressed as a percentage of MAC (C5b-9) formation and calculated using the formula: (Sample-NC)/(PC-NC) x100. IC_{50} values were analysed in GraphPad Prism with inhibitor versus response analysis employing a four-parameters variable slope fit.

Binding affinity studies using Biolayer Interferometry

Measurements of binding affinities for mini-CR1, FHL-1, and FH to C3b were performed using an Octet RH384 Biolayer Interferometry (BLI) system (Satorius, Germany). Streptavidin biosensors were prepared by immobilizing human C3b (Merck, UK) that has been biotinylated using an EZ-Link Sulfo-NHS Biotin kit (ThermoScientific, UK) according to the manufacturer's instructions. Then mini-CR1, FHL-1 and FH were separately passed over the C3b coated sensors to allow calculation of association and dissociation rate constants. Experiments were performed at 25°C and in PBS with 0.2% (v/v) surfactant P20 (PBST). Biotinylated C3b was coated onto Streptavidin biosensors in a total volume of 200 µl/well, at a concentration of 1 mg/ml for 10 minutes. Each protein of interest (i.e. mini-CR1, FHL-1 or FH) was tested separately, in triplicate, in concentrations ranging from 50-1000 nM with the following data acquisition settings: loading, 600 sec; baseline, 150 sec; association, 600 sec; and dissociation 600 sec. After subtraction of control (blank) sensor values for each condition, association

and dissociation rate constants were determined by global data analysis using the Octet® data analysis software.

Ussing Chamber Diffusion

In each experiment a 5 mm diameter disc of macular Bruch's membrane obtained from human donor eyes without macroscopic evidence of AMD or other macular pathology was mounted in an Ussing chamber, forming a barrier between two identical compartments as shown in Figure 3D. Both sides of the Bruch's membrane were subjected with a 5 min wash of 2 mL PBS at room temperature. PBS (2 mL) or purified proteins recombinant mini-CR1, FH, FB or FI in PBS (100 µg/mL, 2 mL volume) were added to the sample chamber. After 1 min, if no leaks occurred into the second compartment, 2 mL PBS was added to the diffusate chamber. This set up was maintained at room temperature for 24h with gentle stirring in both compartments to prevent protein diffusion gradients. Subsequently, 20 µl samples from each chamber were analyzed by gel electrophoresis. Gels were either stained with Instant Blue stain or subjected to Western blotting. To calculate the percentage of protein in the sample or diffusate chambers, band densities in the Instant Blue stained SDS gels were measured using ImageJ64 (version 1.40g; http://rsb.info.nih.gov/ij). The average intensity of these bands, over five separate experiments, were compared to the density of control bands that represent 100% loaded protein (i.e., 20 µl of 100 µg/ml). The calculated percentage protein was then plotted ± SD. For checking cofactor activity of mini-CR1 after diffusion, C3b breakdown assay was performed using 2 µg of C3b and 0.04 µg FI with either FH or 10 µL sample taken from diffusate chamber as described above.

Transduction of RPE cell lines and analysis of expression

For the transduction of ARPE19, hTERT-RPE1, and primary RPE cells, 90,000 each of these cells were plated in 24-well plates and after 24 hrs cells reached 50% confluency i.e. 120,000 cells/well. Once the cells reached 50-60 % confluence, the culture medium from cells was removed and 500 µL of medium containing either CTx001 or null vector rAAV were added, at multiplicity of infections (MOIs) ranging 10,000, 50,000 and 100,000. These viral transduction rates experiments were performed in triplicate. After 72hrs post-transduction, media was collected, PMSF was then added (1.5 mL media + 1.2 µL PMSF) to the conditioned media which was subsequently analyzed by Western blotting. Additionally, cells were collected for qRT-PCR analysis to further evaluate the transduction efficiency.

Western blotting was performed to validate the transduction efficiency of rAAV2 expressing mini-CR1 (CTx001) in RPE cell lines and for evaluating cofactor activity of mini-CR1 after diffusion through Bruch's membrane (from Ussing chamber experiment). For Western blotting, a wet transfer apparatus was used applying 20V for 1 hour, and the protein were transferred onto PVDF membrane in a buffer containing 25 mM Tris, 192 mM glycine, 10% methanol. PVDF membranes were blocked in

10% milk, 0.2% BSA in PBS overnight at 4°C before addition of polyclonal rabbit anti-human mini-CR1 antibody (1:500 diluted, produced in-house) against mini-CR1 protein and anti C3 antibody (clone 1H8, Abcam, UK) for detecting C3-breakdown products for 1 hour at RT. Membranes were washed three times for 30 min each in PBS-T (0.2% tween 20 in 1x PBS) before the addition of HRP conjugated secondary antibody for 1 hour at RT. Membranes were washed three times for 30 min each in PBS-T (0.2% tween 20 in 1x PBS) before the addition of 40:1 ECL Plus Western blotting substrate (Pierce, Thermo Fisher) for 1 min in the dark and then the Fusion imager was used for detection of protein bands.

For rtPCR RNA was extracted from rAAV-mini-CR1 construct transduced and untransduced ARPE19 cells, hTERT-RPE cells, and primary RPE cells using the Isolate RNA Mini Kit (Bioline, catalogue number BIO-52072), and from the posterior cups of rats using Total RNA Purification Plus Kit (Norgen Biotek) following the respective manufacturer's protocol. Isolated RNA was quantified using nanodrop and converted into cDNA using the Superscript IV Vilo Master Mix (cat no. 11756050, Invitrogen). Quantitative PCR was performed using custom-designed specific mini-CR1 Pair 1 FAM-labelled TaqMan probes, (Forward Primer Sequence: AGGACGTGTGCAAGAGAAAG, reverse primer sequence: GTGGTGCAGCTGTAGTTGAT, Probe sequence: CTCCTGATCCTGTGAACGGCATGG). In brief, 10-100 ng of cDNA was suspended in a reaction mix consisting of 1 µl of Mini-CR1 Pair1 TaqMan probe (target) and either 1 µl of VIC labelled TaqMan GAPDH probe (Hs02758991_g1) or 18s probe as control, 10 µl of 2x reaction TaqMan™ Universal Mastermix II, no UNG (Applied Biosystems™, ThermoFisher Scientific, cat no. 4440040), in a final reaction volume of 20 µl in 96 well plates. The qRT-PCR samples were run in triplicate in an ABI 7500 Real-Time PCR system (Applied Biosystems) under the following thermal cycling conditions: initial denaturation at 95°C for 10 min, followed by 40 cycles of denaturation at 95°C for 15 sec and annealing/extension at 60°C for 1 min. Gene expression levels were normalized to *GAPDH* or 18s expression and the relative expression was determined by the ΔΔCt method.

Human iPSC-RPE culture and transduction

Human induced pluripotent stem cell (iPSC) derived RPE cells (iPSC-RPE, Phenocell, France) were cultured according to manufacturer's instructions. Briefly, cells were thawed and then seeded onto Matrigel® coated (final density of Matrigel 8-10 µg/cm^2) transwell-12 inserts at a density of 100,000 cells/cm^2. The cells were then grown for 28 days in culture medium containing 70% DMEM, high glucose, 30% Ham's F12 Nutrient Mix, 2% B-27® Supplement, 1% Antibiotic-Antimycotic; by which time the cells acquired their characteristic polygonal morphology and were pigmented. This maturation was confirmed also with ICC staining for occludin and ZO-1 proteins. Transwells containing iPSC-RPE (each ~112,000 cells) were transduced with one of three doses: low, at 9x10^8 vg/transwell

(~8,000 MOI); medium, at $4.5x10^9$ vg/transwell (~40,000 MOI); or high, at $2.2x10^{10}$ vg/transwell (~200,000 MOI). An empty capsid, null control was also included at $3.4x10^{10}$ vp/transwell. The transduction solutions, 0.8 ml apical side (insert) and 0.8 ml basolateral side (bottom well), total 1.6 ml per cell system, were incubated for 3 days. After 3 days of transduction, apical (~0.7 ml) and basolateral (~0.7 ml) media samples were collected in tubes containing protease inhibitor cocktail on 3, 7, 10, 14, 17 and 21 days. After the media sample collection, fresh cell culture media were added in the apical and basolateral side of the cells and samples were frozen and stored at -70°C. These experiments were repeated to test consistency between batches of cells.

Immunostaining of occludin, ZO-1 and mini-CR1

Methanol fixed cells were washed with 1x DPBS and blocked with 10% (v/v) normal goat serum in DPBS for 20 min. Rabbit anti-occludin, rabbit anti-ZO-1 and a custom anti-human mini-CR1 rabbit monoclonal antibody (FairJourney Biologics, Porto, Portugal, clone FJ2309) primary antibodies were diluted (1:100) in 0.5% normal goat serum in 1x DPBS and incubated at 2-8°C overnight. Cells were washed carefully with 0.5% normal goat serum in 1x DPBS and immunoreacted with anti-rabbit secondary antibody AF488 (1:500) for 90 min. Cells were washed carefully with 0.5% goat serum in 1x DPBS and nuclei were stained with 4',6-Diamidino-2-phenylindole dihydrochloride (DAPI, 1:5000) for 5 min. Cells were cut-out with the membrane and placed on microscope slide, mounted with Fluoroshield and cover slipped. The no primary antibody control was included in the experiments. Signals from ZO-1, occludin and mini-CR1 were captured at 40X in the green channel and DAPI in the Blue channel using a Leica K5 camera (Leica Microsystems GmbH) attached to a Leica Thunder Imager 3D Tissue (DM6B-Z, Leica Microsystems GmbH). The whole system was controlled by the LAS X software (v. 3.7.5.24914, Leica Microsystems GmbH).

Mesoscale [MSD] electrochemiluminescence Mini-CR1 and C3b-iC3b quantitation assays

MSD assays were performed to assess Mini-CR1 and C3b-iC3b in ARPE-19 cell lysate or culture medium supernatant samples. Standard 96-well MSD plates were coated overnight at 4°C with the appropriate capture antibodies diluted to 8 µg/mL in 1× PBS: a custom anti-human mini-CR1 rabbit monoclonal antibody (clone FJ225_mAb02F09) for Mini-CR1 assessment, and a goat polyclonal anti-human C3 antibody for C3b-iC3b measurement [Invitrogen]. Following incubation, plates were blocked with 5% BSA in 1×PBS on a plate shaker (750 rpm) for 90 minutes at room temperature, then washed three times with 1× PBS. Standard curves were prepared using purified human Mini-CR1 (125-0 ng/mL), and C3b (500–0 ng/mL), all diluted in 500 µg/mL BSA in 1×PBS. Blank wells contained 500µg/mL BSA in 1× PBS. Samples and standards were added to the plate, sealed, and incubated on a plate shaker (750rpm) for 90 minutes at room temperature, followed by three washes with 250µL 1× PBS. For

detection, monoclonal antibodies were sulfo-tagged using manufacturers' protocol: a custom anti-human mini-CR1 rabbit monoclonal antibody (clone FJ225_mAb03H10, 2 µg/mL), and an anti-human C3b-iC3b1 antibody (clone 1H8, 1 µg/mL), all diluted in 500µg/mL BSA in 1× PBS. Plates were incubated with detection antibodies for 60 minutes at room temperature, followed by four washes with 1× PBS-Tween 20 (0.05%), and a final wash with 1× PBS. Just before analysis, MSD reading buffer was prepared by diluting MSD Reading Buffer 1:1 in DI water then added to the plate and immediately analyzed using the Meso QuickPlex SQ reader. Data were processed using MSD Discovery Workbench software, applying standard analysis parameters.

C9 Neoepitope staining

The mouse anti-human TCC C9 neoepitope antibody; clone aE11 (HyCult) was used to detect MAC on iPSC derived RPE cells. Supernatants were carefully removed and wells were washed with PBS. After discarding PBS, absolute ethanol was added to each well and incubated for 10min to fix the cells. The wells were subsequently washed twice with PBS. Following washing blocking buffer—composed of 10% normal donkey serum in 5% bovine serum albumin—was added to each well and incubated for 1h at room temperature to prevent non-specific antibody binding [secondary antibodies are raised in donkey]. The blocking buffer was then discarded, and the wells were incubated overnight at 4°C with primary antibody solution [mouse anti-human TCC C9 neoepitope antibody; clone aE11; HyCult]. Following overnight incubation, wells were washed three times with PBS. Next, secondary antibody [donkey anti-mouse Alexa-fluor 488] was added to each well and incubated for 75min at room temperature. Next, secondary antibody solution was removed, and wells were washed three times with PBS for 10min. Finally, the wells were covered with Fluoroshield Mounting Medium [containing DAPI to counterstain nuclei].

C9 Neoepitope staining imaging and quantitation

For consistency, multiple 10x images per well were captured [eVOS instrument], with images taken from the top, middle, left, and right areas of each well. Immunofluorescence signal quantitation was performed using ImageJ. Each imaging channel from the 10x images was transformed into 8-bit format. Background staining was subtracted individually for each image, ensuring consistent baseline correction across all fluorescence channels. Staining quantitation was performed using ImageJ's thresholding function to enhance consistency. Image acquisition settings varied depending on the channel: for DAPI, the light intensity was lower than 10 and exposure ranging between 9 and 90ms, while for 488 [C9 neoepitope], the light intensity was set to 100, with exposure at 350ms. Digital gain was not used to reduce quality artefacts. Upon image input, channels were split, and the 488 channel, corresponding to C9 neoepitope staining, was used for analysis. The image was processed in 8-bit

format, and background removal was performed by disabling smoothing with a rolling ball radius of 50 pixels. After background subtraction image threshold was adjusted to 20 for all images to provide for consistent and comparable quantitation across images, conditions and plates.

***In vivo* rat studies**

The *in vivo* rat studies were performed by Powered Research, USA. Animals were anesthetized and placed under a stereoscope (Leica Microsystems) and a drop of iodine was applied on the cornea and allowed to spread evenly (Minims Povidone Iodine 5%, Laboratoire Chauvin S.A.). A sclerotomy in the temporal side was performed with a 30G needle in order to expose the choroid. The cornea was punctured to reduce the intraocular pressure. A microsyringe (Hamilton Bonaduz AG) was filled with rAAV2 particles and introduced into the subretinal space through the exposed choroid. The solution was injected into the subretinal space for 10 sec, and the needle was kept in place for an additional 30 sec before being removed. The success of the injection was confirmed using in vivo SD-OCT imaging (Bioptigen Envisu R2210. Bioptigen Inc./Leica Microsystems). Chloramphenicol ointment was applied after the injection (Oftan Dexa-Chlora, Santen Oy). CNV induction was performed 28 days after administration of rAAV2 particles. Rats were anesthetized, and pupils were dilated by topical instillation of 1% tropicamide (Tropicamidum WZF 1%: Polfa S. A., Poland or Mydriacyl, s.a. Alcon-Couvreur n.v., Belgium). Carbomer gel (Lakripos, UrsaPharm, Germany) was applied to the right eye (OD) and a coverslip was used to applanate the cornea. Three laser lesions were placed around the optic nerve head of the right eye (OD) using a 532 nm diode laser (Lumenis®, Novus Spectra) and using the following standardized settings: spot size: 100 µm; power: 100 mW; time: 100 ms.

Choroidal flat-mounts were incubated overnight with fluorescein-labeled isolectin B4 (from Griffonia simplicifolia lectin I) (dilution 1:200) to detect neovascularization and subsequently incubated overnight with rabbit C5b-9 antibodies (dilution 1:350) to detect MAC (membrane attack complex) deposition. Following this, choroids were incubated for an additional 3 hours with goat anti-rabbit antibodies conjugated with AlexaFluor 594 fluorescent marker to visualize primary C5b-9 antibody. After each incubation step, samples were thoroughly washed for 10 mins with TBS (Tris-buffered saline) buffer to remove any unbound antibodies and other contaminants. Samples were then mounted using Fluoroshield. Slides were imaged using a Leica B6 microscope (Leica Microsystems). The stained areas were outlined and measured using a validated protocol in the image processing software FIJI. Each image was evaluated individually, by identifying lesion and thresholding.
