# Supplementary material for: CTx001 for Geographic Atrophy: A Gene Therapy Expressing Soluble, Truncated Complement Receptor 1 (Mini-CR1)
Source: Ophthalmol Sci. 2025 Oct 21;6(1):100980. doi: 10.1016/j.xops.2025.100980 (PMC12689202; doi:10.1016/j.xops.2025.100980)
Supplement: Supplemental Figure 1 [file mmc2.pdf]

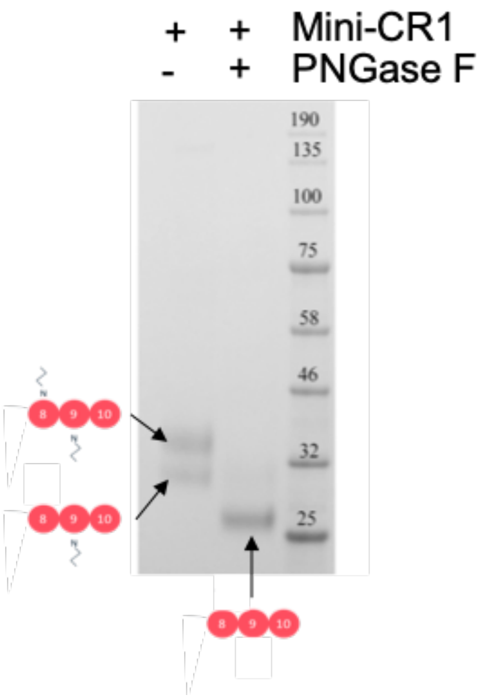

Supplementary Figure 1. Glycosylation of mini-CR1 and its ability to generate C3dg. A) Purified recombinant mini-CR1 runs as two separate bands on a reducing SDSPAGE 4-12% gel and visualised with Coomassie Blue stain. Both bands resolve into a single band after removal of N-linked glycosylation by treatment with PNGaseF. Data shown represents three independent experiments.
