# Supplementary material for: CTx001 for Geographic Atrophy: A Gene Therapy Expressing Soluble, Truncated Complement Receptor 1 (Mini-CR1)
Source: Ophthalmol Sci. 2025 Oct 21;6(1):100980. doi: 10.1016/j.xops.2025.100980 (PMC12689202; doi:10.1016/j.xops.2025.100980)
Supplement: Supplementary Figure 2 [file mmc3.pdf]

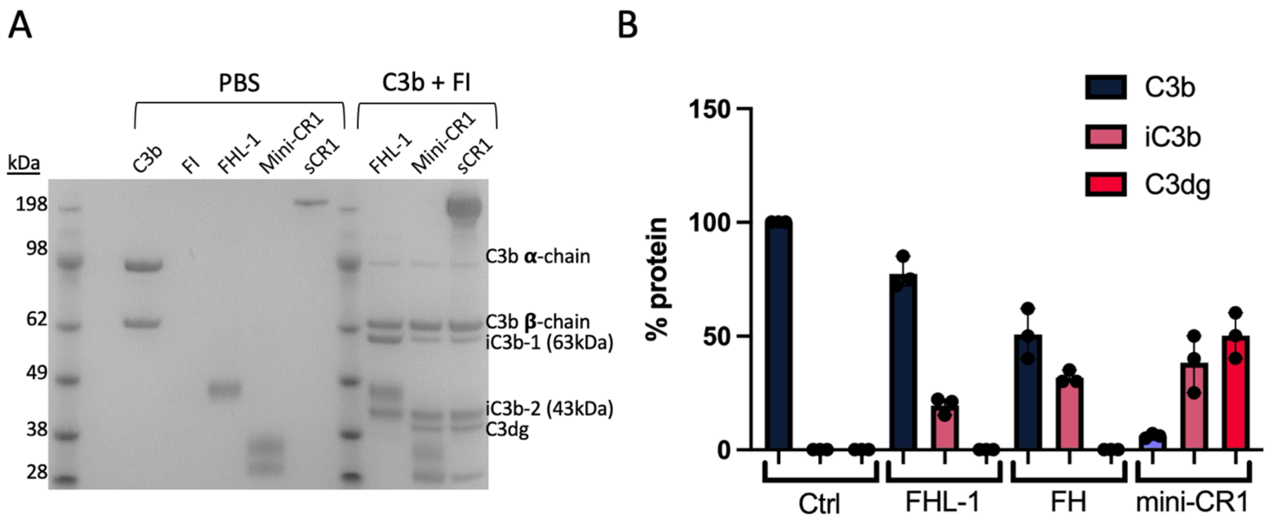

Supplementary Figure 2. Mini-CR1 drives C3b breakdown beyond iC3b. A) Analysis of the breakdown of C3b in the presence of FI either alone (ctrl) or in the presence of different cofactors at equimolar concentrations: FHL-1, FH and or mini-CR1. Experiments were performed in fluid phase and resolved by SDS-PAGE gel electrophoresis. Band densitometry was performed and the conversion of C3b into iC3b and C3dg is reported as total percentage protein (i.e. compared to band density of C3b alone). Only bands corresponding to C3dg were found where mini-CR1 was the FI co-factor used in the experiment (see Figure 3A). B) C3b cleavage assays performed with Factor I (FI) in the presence of: FHL-1; mini-CR1; and full length CR1 cofactors at a fixed dose of 1μM. Note that FHL-1 cannot support cleavage beyond the iC3b intermediate.
