# Supplementary material for: CTx001 for Geographic Atrophy: A Gene Therapy Expressing Soluble, Truncated Complement Receptor 1 (Mini-CR1)
Source: Ophthalmol Sci. 2025 Oct 21;6(1):100980. doi: 10.1016/j.xops.2025.100980 (PMC12689202; doi:10.1016/j.xops.2025.100980)
Supplement: Supplementary Figure 3 [file mmc4.pdf]

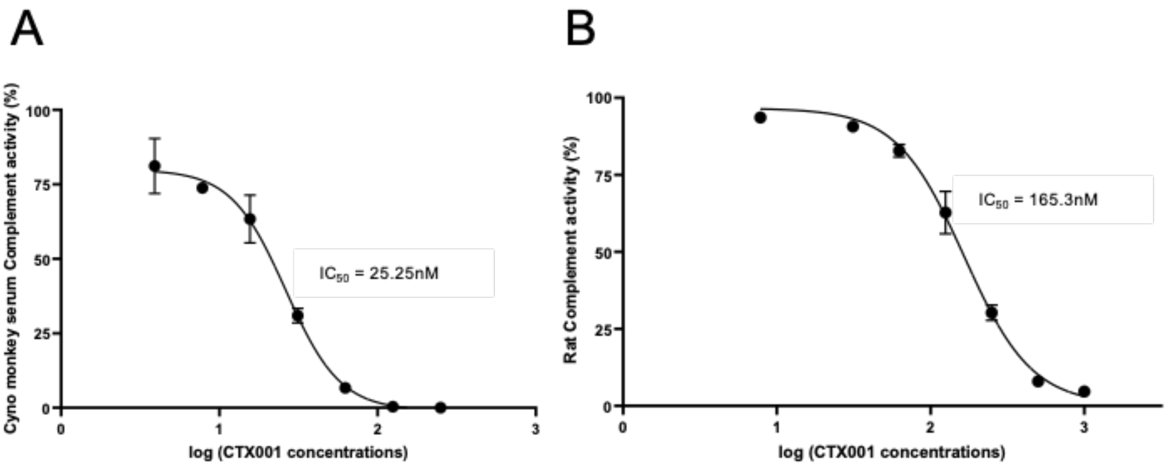

Supplementary Figure 3. IC50 analysis for mini-CR1 in NHP and rat serum. By way of comparison, purified recombinant mini-CR1 was tested for efficacy in inhibiting the alternative pathway of complement in the serum of NHP (A) or rat (B)using commercially available Weisslab assays.
