# Supplementary figures and images for: CTx001 for Geographic Atrophy: A Gene Therapy Expressing Soluble, Truncated Complement Receptor 1 (Mini-CR1)
Source: Ophthalmol Sci. 2025 Oct 21;6(1):100980. doi: 10.1016/j.xops.2025.100980 (PMC12689202; doi:10.1016/j.xops.2025.100980)

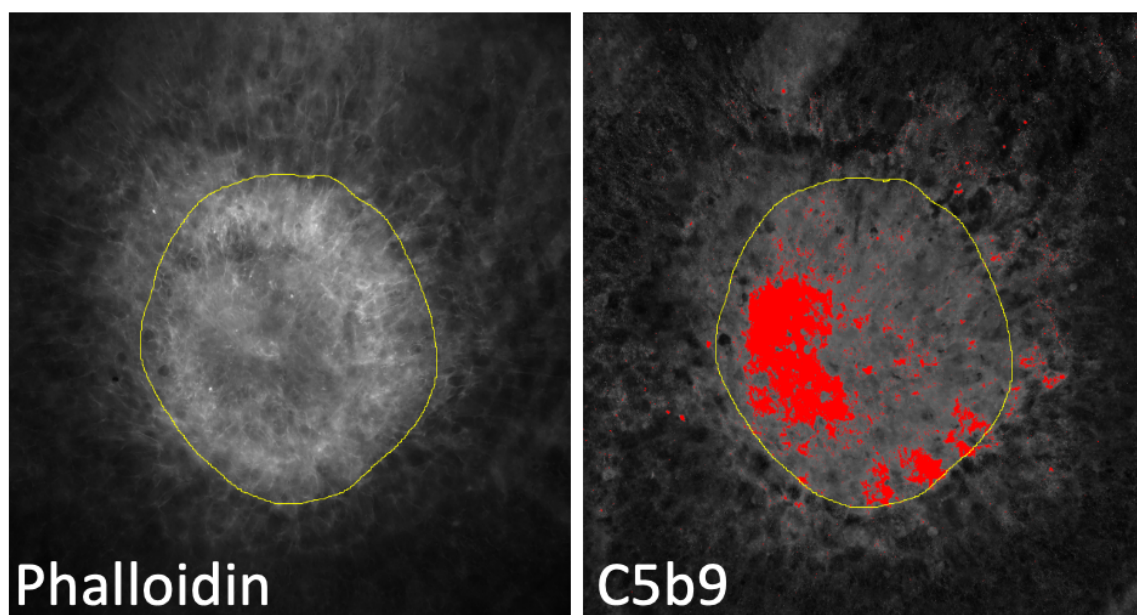

**Supplemental Figure 4. Quantitative image analysis of MAC staining within the laser lesion ROI.**

Supplement: Supplementary Figure 4 [file mmc5.pdf]
