# Supplementary material for: CTx001 for Geographic Atrophy: A Gene Therapy Expressing Soluble, Truncated Complement Receptor 1 (Mini-CR1)
Source: Ophthalmol Sci. 2025 Oct 21;6(1):100980. doi: 10.1016/j.xops.2025.100980 (PMC12689202; doi:10.1016/j.xops.2025.100980)
Supplement: Supplementary Table 1 [file mmc6.pdf]

Supplementary Table 1: Experimental groups used in the *in vivo* rat laser-induced CNV study

Group	Treatment	# Animals / # injected eyes (OD injected)	CNV(OD)	Dose (vg/eye)	Dosing Volume	FA and OCT	Flat mount (OD eyes)
1	Null	9 rats/18 eyes	Yes	$5x10^9$vg/eye, subretinal	2.5 µL	At day 0 and day 1	N=10 eyes/grp for flat mounts N=8 eyes/grp eyecup lysates
2	CTx001 Low	9 rats/18 eyes	Yes	$1x10^8$vg/eye, subretinal	2.5 µL	At day 0 and day 1	
3	CTx001 High	9 rats/18 eyes	Yes	$5x10^9$vg/eye, subretinal	2.5 µL	At day 0 and day 1	
